# The ordinary work environment increases symptoms from eyes and airways in mild steel welders

**DOI:** 10.1007/s00420-015-1041-2

**Published:** 2015-03-06

**Authors:** Lena S. Jönsson, Håkan Tinnerberg, Helene Jacobsson, Ulla Andersson, Anna Axmon, Jørn Nielsen

**Affiliations:** 1 Division of Occupational and Environmental Medicine, Lund University Hospital, 22185 Lund, Sweden; 2 Medical Statistics and Epidemiology Unit, R&D Centre Skåne, Skåne University Hospital, Lund, Sweden

**Keywords:** Work-related symptoms, Diary study, Risk factors, Medical surveillance

## Abstract

**Purpose:**

We aimed to follow diary-registered symptoms from eyes and airways in mild steel welders and relate them to different exposure measures. Furthermore, we would clarify the influence of possible effect modifiers.

**Methods:**

Non-smoking welders with (*N* = 74) and without (*N* = 32) work-related symptoms the last month were enroled. Symptoms and work tasks each day for three two-week periods during 1 year were obtained. Respirable dust (RD) was measured 1 day each period for each worker. The personal daily exposure was assessed as: (1) days at work, (2) welding time and (3) estimates of RD from welding and grinding, calculated from diary entries and measurements.

**Results:**

Only 9.2 % of the particle measurements exceed the Swedish occupational exposure limit (OEL; 5 mg/m^3^). Days at work increased the risk of symptoms studied: eyes: 1.79 (1.46–2.19), nasal: 2.16 (1.81–2.58), dry cough: 1.50 (1.23–1.82) and wheezing and/or dyspnoea: 1.27 (1.03–1.56; odds ratio, 95 % confidence interval). No clear dose–response relationships were found for the other exposure estimates. Eye symptoms increased by number of years welding. Nasal symptoms and dry cough increased having forced expiratory volume in first second below median at baseline. Wheezing and/or dyspnoea increased in winter, by number of years welding, having a negative standard skin-prick test and having a vital capacity above median at baseline.

**Conclusion:**

The current Swedish OEL may not protect welders against eye and airway symptoms. The results add to the evidence that welders should be offered regular medical surveillance from early in the career.

## Introduction

Welding fumes may induce reactive oxygen species (Li et al. 2004; Brand et al. 2010; Leonard et al. 2010) and be hazardous to eyes and airways eliciting both acute effects and manifest disease (Antonini 2003). Adverse effects in the airways may be caused by gases as well as particles in the welding fumes (Antonini 2003), although particles may be the more prominent exposure (Schoonover et al. 2011; Hedmer et al. 2014). The size of the particles influences the deposition in the airways and the health effects (Oberdörster et al. 2005; Sturm 2010). Thus, it has been claimed that particles in the nano-range may be of special interest for the effects on the peripheral airways (Oberdörster et al. 2005). Particles in the welding fume are small, from 20 to 100 nm in diameter (ultrafine particles; Zimmer and Biswas 2001). However, welders may also be exposed to particles from other sources, e.g. when grinding the welded material. At grinding, larger particles are generated although a high fraction of them is respirable (Zimmer and Maynard 2002).

However, the findings of health effects are not consistent as the number of studies showing no effects or health effects only to special welding procedures are considerable (Antonini 2003; Lillienberg et al. 2008). The reasons for the diversities are many. Methodological problems are considerable at such studies. Furthermore, the exposure is complex; various materials and welding methods are employed, and personal protection varies. Daily exposure may vary and may occasional give rise to symptoms. It is reason to believe that frequent occasional symptoms may increase the risk of manifest disease in the airways (Rosenhagen et al. 2012) although firm knowledge does not exist (Francis et al. 2007). Furthermore, to our knowledge, the association between such symptoms and exposure at least in the low exposure range is lacking and so is the meaning of modifying factors. As hundred thousands of workers worldwide are exposed to welding fumes (Antonini et al. 2003), it is important to clarify the health risks in this environment.

In a panel of mild steel welders, we aim to follow diary-registered symptoms from eyes and airways and relate them to different exposure measures. These are presence at work, welding time (WT), supposed to reflect the exposure to small particles, and an estimate of exposure to respirable particles supposed to reflect exposure to larger particles, e.g. from grinding. Furthermore, we aim to study the influence of possible effect modifiers.

## Materials and methods

### Study population

We have earlier established a cohort of 382 welders, mainly working with non-coated mild steel in southern Sweden (Hedmer et al. 2014). The welders produced heavy vehicles, such as dumpers, trucks, asphalt rollers and railway wagons, as well as stoves, windmill towers and wheel containers. From workshops with more than eleven welders, all non-smoking subjects who had reported work-related symptoms from the upper and/or lower airways the last month in a screening questionnaire were invited to the panel. Two welders refused. Thus, 74 workers were included. Of these, 52 had experienced symptoms from the lower airways and 22 only from the upper airways. Thirty-two welders without symptoms the last month, who were matched to the symptomatic welders with regard to factory, age and atopy, were also included. Ex-smokers were included if their current tobacco-free period was more than 5 years. Seven subjects who had declared in the screening questionnaire that they were never smokers turned up to be former smokers at the medical examination. Thus, the total panel consisted of 106 mild steel welders of whom 23 were former smokers (pack-year = 2.2, 0.3–15; median, min–max). The fraction of self-reported lifetime prevalence of asthma in the panel was equal to that of the rest of the cohort (9.9 vs. 9.8 %). Chronic bronchitis (see below) was less common in the rest of the cohort than in the panel (16.3 % vs. 22.3 %). No significant differences were noticed with regard to atopy and symptoms the last month (Table 1). The study subjects gave their informed written consent, and the Regional Ethical Committee of Lund University approved the study.

**Table 1:** Age, years with welding work [median, (min–max)], ever smoking, atopy, asthma, chronic bronchitis, medicine use and symptoms the last month [numbers (%)] at the screening examination by questionnaire in the panel group and in the rest of the welders

	Panel *N* = 106	The rest of welders *N* = 276	*p* value
Age (years)	37 (20–63)	41 (19–64)	0.008^a^
Working with welding (years)	12 (0.5–44)	16 (0.2–45)	0.005^a^
Ever smoking	16 (15.2)	194 (71.9)	<0.001^b^
Atopy	24 (23.1)	63 (23.0)	1.0^b^
Asthma	10 (9.9)	26 (9.8)	1.0^b^
Chronic bronchitis	23 (22.3)	43 (16.3)	0.18^b^
Medicine use	16 (15.1)	38 (13.8)	0.74^b^
Symptoms			
Eyes	36 (34.6)	110 (41.0)	0.29^b^
Nose	60 (57.7)	127 (46.9)	0.066^b^
Cough	32 (30.8)	83 (30.2)	0.90^b^
Wheezing/dyspnoea	21 (20.2)	57 (20.8)	1.0^b^

^a^Mann–Whitney *U* test

^b^Fisher’s exact test

### Study design

The panel went through a medical examination (see below). Thereafter, the members filled in a diary for three two-week periods during the year: (1) the first 2 weeks after the summer holiday, (2) during wintertime and (3) during springtime. Eighty-one welders (76.4 %) filled in the diary during all three periods, 17 welders (16.0 %) during two periods, and eight welders (7.5 %) during one period. The diary contained questions about symptoms and work tasks. During each diary period, exposure measurements in the work place were carried out (see below). Based on the measurements and the diary information, two exposure estimates were calculated for each individual for each day. These estimates were then related to the corresponding daily symptoms.

### Medical examination

A structured interview examined working and medical history, including symptoms from the eyes (running, itching, burning and/or dry eyes), upper airways (blocked, running, itching and/or sneezing nose) and lower airways (attacks of wheezing breath and/or dyspnoea, attacks of dry cough) during the last year and their relationship to work, smoking habits and atopy by history (Littorin et al. 2000). Chronic bronchitis was defined according to Medical Research Council (1965). Atopy by history was defined as a history of hay fever, asthma and/or eczema during childhood and/or adolescence. Work-related symptoms were those associated with work, i.e. recovery during weekends and vacancies (Ferris 1978).

A physical examination including lung auscultation and a skin-prick test, using a standard panel (ALK Abello A/S, Copenhagen, Denmark) of 12 common allergens [birch, grasses (Phleum prat., Dactylis glom., Arrhenatherum elat.), mugwort, cat, dog, horse, house dust mite (Dermatophagoides pteronyssinus), moulds (Cladosporium herb., Alternaria alt., Aspergillus fum.)], was performed. Furthermore, vital capacity (VC) and forced expiratory volume in the first second (FEV_1_) were recorded using a spirometer (Vitalograph, Buckingham, UK) according to the guidelines of European Respiratory Society (Quanjer et al. 1993) and the results related to reference values (VC% and FEV1%; Berglund et al. 1963). Welders with symptoms from the lower airways went through a methacholine test as earlier described (Larsson et al. 2007).

### Diary

At the end of each day, the welders did a diary regarding (1) symptoms from the eyes and airways as described above, (2) symptoms of common cold and fever and medication used during the day, (3) the total number of hours worked, work task (including welding or grinding time) and welding method applied and (4) the number of operations performed and the time consumed for each operation. Also, use of personal protective equipment (PPE) and local ventilation was obtained.

### Air sampling measurements

Personal exposure measurements for ozone and respirable dust (RD) were taken during one full day during each of the three periods of measurements (Hedmer et al. 2014). The average sampling time was 6.2 h. Ozone was measured according to OSHA method ID-214 (OSHA 2008). The measured levels were low (0.03 mg/m^3^ geometric mean; Hedmer et al. 2014) and were not further analysed. Respirable dust was measured with filters placed in RD cyclones (BGI Inc, MA, USA) connected to portable pumps. The levels of RD varied between 0.08 and 38.3 mg/m^3^. The median was 1.5 mg/m^3^, and 24 (9.2 %) of a total of 262 samples were above or at the current Swedish occupational exposure limit (OEL; 2011). The samplings were performed in the breathing zone, but outside the PPE. In the case when air-fed helmets were used, this was compensated for in the exposure model (see below). The detailed exposure measurements can be found in Hedmer et al. (2014).

### Exposure measures

Two different uncorrelated (*R*_s_ = 0.09) estimates for the welding fume exposure were used: (1) actual time spent welding each day as extracted from the diaries, henceforth referred to as WT, and (2) estimated exposure for RD each day (see below).

### Exposure estimate for respirable dust

To estimate the exposure to RD for the days when exposure was not measured, information on work tasks extracted from the diary was used in a statistical linear model based on protocols on daily activity and other measures (Wameling et al. 2000). The idea is to predict exposure with variables easier to obtain than exposure measurements and use these variables in a statistic model.

This model was built from observations (*N* = 262) from those workers for whom we had both data on work tasks and measured dust data. As these workers each could have up to three measurements, the mixed model for repeated measures in SPSS (version 15.0) was used to estimate a suitable model. Estimates were obtained by the restricted maximum-likelihood method. The Schwarz’s Bayesian Criterion suggested that an autoregressive covariance structure should be used in the model. As the measurements of RD were skewed, they were transformed using the natural logarithm before inclusion in the model.

The independent variables in the model were the fraction of the work day spent welding with different techniques (metal inert gas (MIG), metal active gas (MAG), tungsten inert gas (TIG), manual metal arc (MMA), welding with powder (submerged arc welding) and supervision of robot welding), as well as the fraction of the work day spent grinding, and the present company. The estimated dust was calculated accordingly. First, the following formula was used to combine the exposure caused by the different welding methods:

$$z = 0.369{\text{MAG}} + 0.577{\text{MIG}}{-}0.25{\text{MMA}}{-}1.489\,{\text{powder}}{-}0.291\,{\text{robot}} + 0.547{\text{TIG}} + 0.214\,{\text{welding}}$$

Welding represents the fraction of the day working with welding irrespective of method. The effect of grinding was added in the same way (see below). Finally, the use of PPE was taken into account on the basis of measurements taken inside and outside PPE, such that only a third of the estimated RD was used for times when the worker had been using

PPE:

$$y = 0.08300 + \frac{{x_{1} \times z}}{3} + \left({1 - x_{1}} \right) \times z + \frac{{x_{2} \times 0.673\;grinding}}{3} + \left({1 - x_{2}} \right) \times 0.673\;grinding$$

*x*_1_ represents the part of the WT when PPE was used, *x*_2_ the corresponding for grinding, and *grinding* the fraction of the day spent grinding. Finally, the company (*comp*) was adjusted for when the values were back-transformed to original scale:

$${\text{RD}}_{{\text{est}}} = e\{ y - comp\}$$

*comp* ranged between −1.473 and 1.075 for the 11 companies included.

These estimated levels of respiratory dust are henceforth referred to as estimated exposure. The exposure was estimated for all panel members for all days under study, including those that had actual measurements.

### Statistics

To estimate the association between exposure and symptoms, mixed-model analyses on repeated observations were performed. We used symptom specifications from the diaries as a binary-dependent variable (no/yes) and subject as a random factor. Four different symptoms (eyes, upper airways, dry cough and wheezing and/or dyspnoea) were used as outcomes. Different exposure variables (working day—no/yes, estimated exposure of particles and WT) were tested as fixed effects. The estimated exposure and WT were analysed as categorical variables. The categorical variables were created by trichotomising each exposure for the whole study group at their respective 33rd and 67th percentiles. Cut-offs for the group were set at 1.08 and 1.86 mg/m^3^ for estimated RD and at 4.5 and 7.0 h for WT. The days off were used as reference. The analyses with the categorical variables were performed without the days of illness (e.g. fever or common cold).

Confounders added to the models were age (continuous), any medicine use for the symptom analysed (no/yes) and the number of years working with welding (continuous). As effect modifiers, we tested the period of the year (spring/after summer holiday/winter), skin-prick test (negative/positive), VC% [binary; cut-point median (95 %)], FEV1% [binary; cut-point median (99 %)], the number of years working with welding (binary; short-term welders ≤3 (*N* = 7), long-term welders ≥3 years) and medication relevant for the symptom studied (no/yes). Each effect modifier was analysed with the exposure variable working day by adding the effect modifier and the interaction term (modifier × working day) to the model. The total effect (working day with effect modifier) was obtained by removing the main effect of the working day from the model.

The PROC GLIMMIX procedure in SAS was used. SP(EXP) was chosen as covariance structure to account for the three periods of the year. The welders participated during different weeks within each period. The median weeks for the periods were calculated, and the days were used in the analyses. The estimates were obtained by the residual likelihood subject-specific expansion pseudo-likelihood technique if not otherwise stated. The statistical analyses were performed in SPSS 15.0 for Windows (SPSS Inc., Chicago, IL, USA) and SAS 9.2 for Windows (SAS Institute Inc., Cary, NC, USA), and *p* values below 0.05 were considered as statistically significant.

## Results

There were no differences between the symptom groups regarding age, number of years with welding or lung function, but they differed with regard to skin-prick test positivity (Table 2). Only five of the welders having symptoms from the lower airways reacted significant on the methacholine test indicating none-specific bronchial hyperreactivity. The group of workers who at inclusion to the study reported no airway symptom the last month had some symptoms during the study period, but the prevalence was considerably lower than in the other two groups. In total, the welders reported eye symptoms 9.7 % of the days recorded. The corresponding number for upper airway symptoms was 33 %, for wheezing/dyspnoea 2.6 % and for dry cough 14 %.

**Table 2:** Age, years of welding, skin-prick test positivity, medicine used regularly, symptoms the last year before the study and lung function in subgroups and in the total panel at the medical examination [median (min–max) or *N*, (%)]

	Present airway symptoms^a^			
	No *N* = 32	Lower *N* = 52	Upper, only *N* = 22	Total *N* = 106
Age (years)	38 (22–62)	38 (21–63)	36 (22–53)	37 (21–63)
Welding work (years)	13 (0.4–40)	12 (2–44)	10 (2–26)	12 (0.4–44)
Positive skin-prick test	6 (18.8)	18 (34.6)	6 (27.3)	30 (28.3)
Medicine use	2 (6.2)	7 (13.5)	0 (0.0)	9 (8.5)
Symptoms				
Eyes	7 (21.9)	25 (48.1)	5 (22.7)	37 (34.9)
Running nose	2 (6.3)	32 (61.5)	9 (40.9)	43 (40.6)
Nose stuffiness	1 (3.1)	34 (65.4)	13 (59.1)	48 (45.3)
Sneezing/nose itching	2 (6.3)	30 (57.7)	10 (45.5)	42 (39.6)
Wheezing/dyspnoea	1 (3.1)	36 (69.2)	0 (0.0)	37 (34.9)
Dry cough	2 (6.3)	35 (67.3)	0 (0.0)	37 (34.9)
Spirometry^b^				
VC%	96 (66–108)	93 (70–115)	95 (70–118)	95 (66–118)
FEV_1_%	100 (76–114)	98 (66–129)	100 (79–123)	100 (66–129)

^a^Symptoms the last month

^b^Vital capacity (VC) and forced expiratory volume in the first second (FEV1) as % of predicted

### Exposure estimate for respirable dust

The actual measurements and the estimated data were in agreement as judged by visual assessment (not shown) and justified in the median: measured RD (262 samples) 1.45 mg/m^3^ and estimated (2497 samples) 1.44 mg/m^3^. However, the measured exposure had a larger range compared to the exposure estimate (25th–75th percentile 0.77–2.38 vs. 1.01–1.64).

### Dose–response relationships

**Fig. 1:**
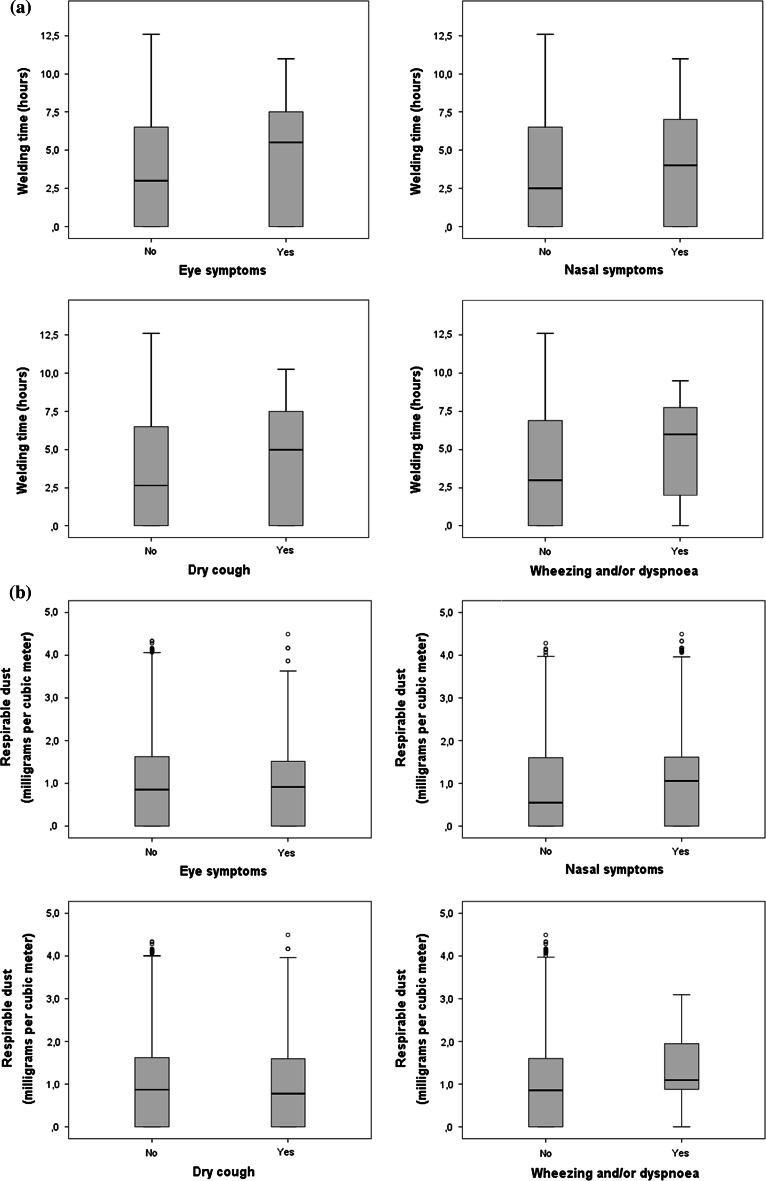
Relation between estimated exposures expressed as welding time (**a**) and respirable dust (**b**) and presence or absence of symptoms, respectively, from eyes, nose, dry cough and wheezing and/or dyspnoea in a *box plot* diagram showing medians and the 25 and 75 percentiles for all estimated exposures during the study periods

When individual relationships between exposure estimates and symptoms or no symptoms, respectively, were studied, welders with symptoms had longer daily WT than those without symptoms (Fig. 1a), whereas exposure only was higher in subjects with nasal symptoms and wheezing and/or dyspnoea when estimated respiratory dust was used (Fig. 1b).

Furthermore, there was a significant increase during working days compared to days off for the four symptoms studied (Tables 3, 4, 5, 6). When the group was trichotomised with regard to exposure levels (WT, estimated respiratory dust), increased risks of all four symptoms were shown in all exposure groups, but straightforward dose–response relationships were generally not demonstrated. Thus, the highest exposure group often had a lower risk than the medium exposed group (Tables 3, 4, 5, 6).

**Table 3:** Eye symptoms associated with different exposure measures for welders and the influence of possible effect modifiers when days at work are used as measure of exposure

	*N*	Category	OR	95 % CI^a^	Interaction^b^ *p* value
Exposure measures^c^					
Respirable dust^d^	105	Low	2.25	1.68–3.02	
		Medium	1.72	1.30–2.27	
		High	1.46	1.04–2.07	
Welding time^d^	105	Low	1.73	1.30–2.30	
		Medium	2.14	1.61–2.85	
		High	1.77	1.40–2.25	
Working day	105		1.79	1.46–2.19	
Working day with effect modifier (total effect and interaction)					
Period of the year	105	Summer^e^	2.43	1.68–3.51	
		Winter	1.54	1.09–2.17	
		Spring	1.50	1.04–2.16	0.12
Skin-prick test	104	Negative	1.97	1.55–2.50	
		Positive	1.47	0.98–2.22	0.23
VC%	105	<95 %	1.70	1.29–2.24	
		≥95 %	1.90	1.41–2.57	0.59
FEV_1_%	105	<99 %	1.92	1.49–2.47	
		≥99 %	1.58	1.13–2.22	0.37
Welding years	105	<3 years	0.77	0.33–1.78	
		≥3 years	1.88	1.52–2.32	0.043
Medication	67	No	2.84	1.88–4.28	
		Yes	1.31	0.93–1.86	0.0051

Estimates were adjusted for age, medication used and the number of years working with welding

^a^The total effect of working day with effect modifier was obtained by removing the main effect of the working day from the model

^b^Each effect modifier was analysed with the exposure variable working day by adding the effect modifier and the interaction term (modifier × working day) to the model

^c^The reference, no working day, is removed from the table

^d^The days of illness were removed from the analysis

^e^The first 2 weeks after summer holiday

**Table 4:** Nasal symptoms associated with different exposure estimates for welding and the influence of possible effect modifiers when days at work are used as measure of exposure

	*N*	Category	OR	95 % CI^a^	Interaction^b^ *p* value
Exposure measures^c^					
Respirable dust^d^	105	Low	2.39	1.86–3.08	
		Medium	3.18	2.44–4.16	
		High	1.65	1.23–2.22	
Welding time^d^	105	Low	2.31	1.82–2.92	
		Medium	2.62	2.05–3.33	
		High	2.14	1.70–2.71	
Working day	105		2.16	1.81–2.58	
Working day with effect modifier (total effect and interaction)					
Period of the year	105	Summer^e^	1.77	1.29–2.44	
		Winter	2.20	1.63–2.95	
		Spring	2.89	2.12–3.95	0.094
Skin-prick test	104	Negative	2.18	1.76–2.70	
		Positive	2.18	1.58–3.00	1.00
VC%	105	<95 %	2.07	1.59–2.68	
		≥95 %	2.25	1.76–2.86	0.65
FEV_1_%	105	<99 %	2.51	1.99–3.16	
		≥99 %	1.75	1.34–2.28	0.044
Welding years	105	<3 years	2.13	1.08–4.18	
		≥3 years	2.16	1.80–2.60	0.96
Medication	67	No	3.02	2.16–4	
		Yes	1.86	1.36–2.56	0.040

Estimates were adjusted for age, medication used and the number of years working with welding

^a^The total effect of working day with effect modifier was obtained by removing the main effect of the working day from the model

^b^Each effect modifier was analysed with the exposure variable working day by adding the effect modifier and the interaction term (modifier × working day) to the model

^c^The reference, no working day, is removed from the table

^d^The days of illness were removed from the analysis

^e^The first 2 weeks after summer holiday

**Table 5:** Dry cough associated with different exposure estimates for welding and the influence of possible effect modifiers when days at work are used as measure of exposure

	*N*	Category	OR	95 % CI^a^	Interaction^b^ *p* value
Exposure measures^c^					
Respirable dust^d^	105	Low	1.79	1.36–2.34	
		Medium	1.85	1.39–2.47	
		High	1.17	0.83–1.65	
Welding time^d^	105	Low	1.56	1.19–2.05	
		Medium	1.54	1.18–2.00	
		High	1.78	1.38–2.29	
Working day	105		1.50	1.23–1.82	
Working day with effect modifier (total effect and interaction)					
Period of the year	105	Summer^e^	1.83	1.30–2.56	
		Winter	1.32	0.95–1.82	
		Spring	1.40	1.00–1.96	0.34
Skin-prick test	104	Negative	1.67	1.32–2.11	
		Positive	1.21	0.84–1.74	0.14
VC%	105	<95 %	1.63	1.24–2.14	
		≥95 %	1.38	1.05–1.82	0.41
FEV_1_%	105	<99 %	1.87	1.41–2.49	
		≥99 %	1.22	0.92–1.60	0.033
Welding years	105	<3 years	1.82	0.86–3.83	
		≥3 years	1.48	1.21–1.80	0.59
Medication	67	No	2.08	1.47–2.96	
		Yes	1.22	0.90–1.65	0.023

Estimates were adjusted for age, medication used and the number of years working with welding

^a^The total effect of working day with effect modifier was obtained by removing the main effect of the working day from the model

^b^Each effect modifier was analysed with the exposure variable working day by adding the effect modifier and the interaction term (modifier × working day) to the model

^c^The reference, no working day, is removed from the table

^d^The days of illness were removed from the analysis

^e^The first 2 weeks after summer holiday

**Table 6:** Wheezing and/or dyspnoea associated with different exposure estimates for welding and the influence of possible effect modifiers when days at work are used as measure of exposure

	*N*	Category	OR	95 % CI^a^	Interaction^b^ *p* value
Exposure measure^c^					
Respirable dust^d^	105	Low	1.93	1.34–2.78	
		Medium	2.57	1.85–3.56	
		High	1.26	0.95–1.67	
Welding time^d^	105	Low	1.99	1.45–2.72	
		Medium	1.34	1.02–1.77	
		High	1.90	1.43–2.53	
Working day	105		1.27	1.03–1.56	
Working day with effect modifier (total effect and interaction)					
Period of the year	105	Summer^e^	1.70	0.89–3.24	
		Winter	3.22	1.76–5.90	
		Spring	0.80	0.53–1.21	<0.001
Skin-prick test	104	Negative	2.06	1.51–2.80	
		Positive	0.88	0.65–1.20	<0.001
VC%	105	<95 %	0.96	0.72–1.29	
		≥95 %	1.63	1.21–2.19	0.014
FEV_1_%	105	<99 %	2.38	1.75–3.23	
		≥99 % or more	0.39	0.27–0.57	–^f^
Welding years	105	<3 years	0.46	0.23–0.94	
		≥3 years	1.43	1.14–1.78	0.0027
Medication	67	No	2.21	0.94–5.22	
		Yes	1.10	0.86–1.41	0.12

Estimates were adjusted for age, medication used and the number of years working with welding

^a^The total effect of working day with effect modifier was obtained by removing the main effect of the working day from the model

^b^Each effect modifier was analysed with the exposure variable working day by adding the effect modifier and the interaction term (modifier × working day) to the model

^c^The reference, no working day, is removed from the table

^d^The days of illness were removed from the analysis

^e^The first 2 weeks after summer holiday

^f^The estimates were obtained by the maximum-likelihood subject-specific expansion pseudo-likelihood technique (MSPL)

### Symptom modifiers

When possible modifiers of symptoms were studied using days at work as the measure of exposure the winter period of the year, having a negative skin-prick test and VC% above the median before the study significantly increased the risk of wheezing and/or dyspnoea (Table 6). Long-term welders (≥3 years) had a significantly increased risk of eye symptoms and of wheezing and/or dyspnoea (Tables 3, 6). FEV1% below the median increased the risk of nasal symptoms and dry cough (Tables 4, 5). Medication used was inversely associated with the risk of all the symptoms but wheezing and/or dyspnoea did not reach statistical significance (Tables 3, 4, 5, 6).

## Discussion

In this diary study of mild steel welders regarding short-term symptoms from eyes and airways associated with different exposure measures, we found that the welders reported significantly more symptoms from eyes and airways on working days compared to days off although the exposure levels generally were below the Swedish OEL. Welders with symptoms had longer WT the same day than those without symptoms, whereas this was true only for nasal symptoms and wheezing and/or dyspnoea when estimated RD was considered. No straightforward dose–response relationships were found for the two exposure measures. Working ≥3 years as a welder increased the risk of eye symptoms as well as wheezing and/or dyspnoea. Wintertime and having a negative skin-prick test also increased the risk of wheezing and/or dyspnoea. A lower FEV1% increased the risk of symptoms from the airways, whereas strange enough a higher VC% increased the risk of wheezing and/or dyspnoea.

In this study, there are some concerns of methodology which should be considered. Twenty-five welders did not fully complete the diary. A dropout was expected because of the long follow-up time. The dropout was due to personal reasons such as change of work or beginning of education. We have no reason to believe that the participants stopped for health reasons even if we are missing information from some few subjects. Thus, we do not think this dropout may influence the risk assessment to any significant degree.

It is earlier shown that mild steel welders have more symptoms from eyes and airways compared to non-welders (Cotes et al. 1989; Mur et al. 1985; Torén et al. 1999). In this study, we showed that such symptoms clearly increase during working days although the exposure was not extreme compared to the current Swedish exposure limit (OEL, 2011). This indicates that the current OEL may not be sufficient.

Using the two other measures for exposure, we found no clear dose–response relationships for neither. Several questions concerning validity of the exposure estimates and selection of the group should, therefore, be considered.

Regarding the statistical model for respirable particles, measured data and estimated data were in agreement as judged by visual assessment. As expected, the estimates from the model did not include extreme exposure levels, as indicated by a smaller range for estimated exposure compared to measured exposure. This will probably tend to press the dose–response curve to the left. However, this is not likely to explain the lack of the dose–response relationships, as the exposure was trichotomised in the analysis.

Particles in the welding environment are emitted from welding and grinding. Although the particles emitted at welding will aggregate in the welders breathing zone, they are still very small (about 100 nm, Zimmer and Biswas 2001). Therefore, the measure “welding time” may to a higher degree reflect an exposure to ultrafine particles with a larger surface per weight unit, compared to the estimated exposure to particles.

The particles emitted from grinding are somewhat bigger than the welding-derived ones, although a substantial amount of these are in the nano- to the micrometre scale, depending on the substrate (Zimmer and Maynard 2002). The grinding-derived particles may then have a larger impact on the estimated exposure to particles than the welding-derived ones. However, no clear differences between the two exposure estimates were noted.

Exposure to RD may be overestimated for the time grinding as the protection factor used in the estimate was the same as the one used for welding. The estimated protection factor is low due to the fact that when welding, the welders repeatedly lift the protective helmet to examine the welding seam and thus get exposed. When grinding, the PPE is used during longer periods, as it is easier to see the results of the work through the visor, compared to when welding. However, that will be true for all the exposure categories studied and may not affect the dose–response relationships. Furthermore, it will not explain the lack of dose–response relationship for WT.

Ozone and nitrogen dioxide may be present in the welding environment, but they are not considered in our estimates. Nitrogen dioxide was initially measured with direct-reading devices, but as levels were low this measure was excluded from the sampling scheme. Only a few samples showed ozone levels higher than the background (Hedmer et al. 2014). Low exposure levels to ozone and nitrogen oxide in welders’ environment have also been shown by Schoonover et al. (2011). Ozone exposure is probably intermittent, and a few very short peaks would then not have been detected. Such short exposures may elicit symptoms in sensitive subjects, but it is hard to believe that this substantially has influenced the dose–response relationships. Regarding symptoms from the eye injures and arc eyes may explain some of the symptoms. One of the authors (JN) has a personal knowledge to some of the workshops included. The welders are well protected, and such injuries do not happen so frequent that the high rate of symptoms can be explained by such injuries.

The same lack of dose–response relationships was found in another study of welders, although the study design was completely different (Lillienberg et al. 2008). This lack was explained by a selection of symptomatic workers away from the highest exposure area. In the present study, welders were selected to the panel because of their symptoms, either they had frequent or few symptoms in their welding environment. Thus, the panel may consist of subjects who in the present exposure interval had symptoms easy triggered and who were resistant, respectively. However, the two groups were matched with regard to the workplace. So it is hard to understand how a selection could explain the lack of dose–response relationship.

Although the panel consisted of a considerable group of symptomatic welders, manifest diseases in the airways and non-specific hyper-reactivity were infrequent, which also is reflected by the low use of medication, normal lung functions in general and only a few subjects reacting at the methacholine test. It is reason to believe that we are witnesses to the effects of processes in the mucous membranes elicited by the injury from the welding environment and modified by repair processes as described in experimental studies of Oh et al. (2009), Leonard et al. (2010), from our group in some of the symptomatic welders studied separately (Jönsson et al. 2011) and furthermore in a cross-sectional study of mild steel welders by Hoffmeyer et al. (2012). However, there is still a lack of knowledge concerning the dose–response relationships and of the mechanisms.

Interestingly, the risk of wheezing and/or dyspnoea increased already after 3 years of welding. We have earlier shown that welders in aluminium and stainless steel already after more than 2.5 years of welding had an increased reactivity in the small airways compared to welders with a shorter WT (Nielsen et al. 1993). The present study may add to the evidence that regularly medical surveillance is important from the beginning of the welding career.

The risk of wheezing and/or dyspnoea was surprisingly increased in subjects having a negative skin-prick test. Prior to the study, welders having work-related lower airway symptoms quite frequently had a positive skin-prick test and thus were supposed to be more sensitive to irritants. Our findings is, however, in accordance with the results from a newly published Scandinavian population-study regarding exposure to low molecular agents and irritants. Non-atopic subjects were then at the highest risk of new-onset asthma (Lillienberg et al. 2012). The result may indicate that atopy is not a marker of risk in the welding environment. However, different selection mechanisms may have influenced these results.

Having a FEV1% before the study under the median increased the risk of all the airway symptoms studied although most had a lung function well within the normal range. FEV1 is affected at obstructive lung diseases, but there is also an association between rhinitis, even in absence of atopy, and adult-onset asthma (Shaaban et al. 2008); this can explain the association between FEV1 and nasal symptoms. A reasonable interpretation of the present findings may be that frequent symptoms from the airways have affected the lung function, so far without manifest disease. This may further indicate an increased risk of manifest asthma as shown in an earlier study by Puolijoki et al. (1992) who followed a group of subjects examined because of suspected asthma but where the examination could not state that. Those who later developed an overt asthma had a lower FEV1% compared to those without asthma development. Contrary, in the present study, VC% above the median increased the risk of wheezing and/or dyspnoea. This is surprising, but our finding is supported by Kalhan et al. (2010) who followed a group of young adults without asthma during 20 years and found that a lower FEV1 and a higher FVC predicted development of asthma.

## Conclusion

Welders’ eye and airway symptoms increased significantly during working days although exposure was generally below the Swedish OEL. Furthermore, welders with symptoms had longer WT the same day as welders without symptoms. This indicates that the current OEL may not protect the workers from hazardous effects. The symptoms may predict later overt disease. The results add to the evidence that welders should be offered regular medical surveillance from early in the career.
